# Analysis of High-Risk Extramedullary Relapse Factors in Newly Diagnosed MM Patients

**DOI:** 10.3390/cancers14246106

**Published:** 2022-12-12

**Authors:** Xiaoyan Yue, Donghua He, Gaofeng Zheng, Yang Yang, Xiaoyan Han, Yi Li, Yi Zhao, Wenjun Wu, Qingxiao Chen, Enfang Zhang, Zhen Cai, Jingsong He

**Affiliations:** 1 Bone Marrow Transplantation Center, Department of Hematology, The First Affiliated Hospital, School of Medicine, Zhejiang University, Hangzhou 310003, China; 2 Department of Hematology, Sir Run Run Shaw Hospital, School of Medicine, Zhejiang University, Hangzhou 310003, China; 3 Institute of Hematology, Zhejiang University, Hangzhou 310003, China; 4 Zhejiang Laboratory for Systems & Precision Medicine, School of Medicine, Zhejiang University, 1369 West Wenyi Road, Hangzhou 311121, China

**Keywords:** extramedullary multiple myeloma, extramedullary relapse, clinical characteristics, prognosis, risk factors

## Abstract

**Simple Summary:**

Extramedullary relapse of multiple myeloma (MM) is often resistant to existing treatments, and has an extremely poor prognosis. Therefore, early identification of high-risk extramedullary relapse patients has important clinical significance. However, due to the lack of a large prospective clinical study, clinical characteristic evidence for the early identification of patients with extramedullary relapse is still lacking. Our study analyzed the high-risk factors for extramedullary relapse in NDMM patients for the first time, hoping to identify high-risk extramedullary relapse patients as early as possible to take early measures to prevent extramedullary relapse and improve the overall prognosis of NDMM patients.

**Abstract:**

Extramedullary relapse of multiple myeloma (MM) is often resistant to existing treatments, and has an extremely poor prognosis, but our understanding of extramedullary relapse is still limited. The incidence, clinical characteristics, impact on the prognosis of extramedullary relapse, and the risk factors for extramedullary relapse in NDMM patients were analyzed. Among the 471 NDMM patients, a total of 267 patients had disease relapse during follow-up, including 64 (24.0%) patients with extramedullary relapse. Extramedullary relapse was more common in patients with younger age, IgD subtype, elevated LDH, extensive osteolytic lesions, extramedullary involvement, and spleen enlargement at the time of MM diagnosis. Survival analysis showed that extramedullary relapse patients had significantly worse median OS than patients with relapse but without extramedullary involvement (30.8 months vs. 53.6 months, *p* = 0.012). Multivariate analysis confirmed that elevated LDH (OR = 2.09, *p* = 0.023), >2 osteolytic lesions (OR = 3.70, *p* < 0.001), extramedullary involvement (OR = 3.48, *p* < 0.001) and spleen enlargement (OR = 2.27, *p* = 0.011) at the time of MM diagnosis were independent risk factors for extramedullary relapse in NDMM patients. Each of the above four factors was assigned a value of 1 to form the extramedullary relapse prediction score, and the 3-year extramedullary relapse rates of patients in the 0–2 and 3–4 score groups were 9.0 % and 76.7 %, respectively. This study suggested that extramedullary relapse was associated with poor clinical characteristics and poor prognosis in NDMM patients. The extramedullary relapse prediction score model composed of LDH, osteolytic lesions, extramedullary involvement and spleen enlargement has a better ability to predict extramedullary relapse than the existing ISS and R-ISS stages.

## 1. Introduction

Multiple myeloma (MM) is characterized by malignant proliferation of clonal plasma cells in the bone marrow and secretion of a large number of monoclonal immunoglobulins, causing a series of clinical symptoms, such as bone destruction, hypercalcemia, anemia, and renal insufficiency [1]. Although a large number of new targeted drugs, represented by proteasome inhibitors and immunomodulators, have entered the clinic in recent years, the application of autologous hematopoietic stem cell transplantation (ASCT) has significantly improved the prognosis of MM patients [2,3,4,5]. However, MM is still an incurable disease, and almost all patients will eventually relapse and be refractory to treatment.

It is well known that the tumor microenvironment plays an important role in the occurrence and development of MM, and the survival and proliferation of MM cells depend on the signals generated by the bone marrow microenvironment. Therefore, most MM tumor cells are usually confined to bone marrow, but in some cases, malignant plasma cells can also metastasize along the bone marrow compartment, break through the limitation of the bone marrow and bone tissue to form tumorous masses in the adjacent bone site, and even enter the blood circulation and spread to distant tissues to form tumorous masses, which is known as extramedullary multiple myeloma (EMM) [6,7,8]. EMM can appear at the time of MM diagnosis or relapse, and the clinical characteristics of EMM at diagnosis and relapse are significantly different [9,10,11]. In recent years, the incidence of extramedullary relapse has gradually increased due to the improvement of diagnosis and treatment methods, and studies have confirmed that extramedullary relapse is significantly related to the high-risk characteristics and poor prognosis of MM patients, which cannot be significantly improved even in the era of new drugs [10,11,12]. Due to the poor treatment effect and extremely poor prognosis of extramedullary relapse patients, it is necessary to identify high-risk patients with extramedullary relapse as early as possible in clinical practice to take active diagnosis and treatment measures to prevent extramedullary relapse and improve the overall prognosis and survival of MM patients. However, due to the lack of a large prospective clinical study, clinical characteristic evidence for the early identification of patients with extramedullary relapse is still lacking.

Therefore, the primary objective of this study was to analyze the incidence, clinical characteristics, and survival status of EMM patients at the time of relapse, as well as the high-risk factors for extramedullary relapse in newly diagnosed MM (NDMM) patients based on real-world data from 471 NDMM patients, hoping to serve as a reference for the prevention and treatment of extramedullary relapse in the era of new drugs.

## 2. Method

### 2.1. Research Patients

This study was a retrospective analysis, approved by the Ethics Committee of the First Affiliated Hospital of the Medical School of Zhejiang University. A total of 471 patients who were first diagnosed with symptomatic or progressive MM in our center from May 2013 to June 2020 were included. Inclusion criteria for this study were as follows: (1) patients were diagnosed according to the diagnostic criteria of MM established by the World Health Organization (WHO) or the International Myeloma Working Group (IMWG). (2) Patients with complete clinical data were diagnosed and treated in our center, at the time of diagnosis and relapse; (3) M protein was detected in blood and/or urine; and (4) patients received first-line treatment with bortezomib-based regimen in our center and completed at least one course of treatment with efficacy evaluation results. Exclusion criteria for this study were as follows: (1) solitary plasmacytoma (SP), referring to the presence of a single extramedullary lesion, without involvement of bone marrow plasma cells or only a minimal bone marrow involvement (<10%), and the difference between SP and EMM is that SP patients cannot be diagnosed with MM and cannot be attributed to EMM; (2) plasma cell leukemia (PCL), known as malignant plasma cells involving peripheral blood reaching 2000/µL or accounting for ≥ 20% of peripheral blood nucleated cells, is essentially an extreme state of extramedullary extraosseous (EME) [6]; (3) patients who were not treated in our hospital at the time of diagnosis or relapse; and (4) patients who were diagnosed in our center and returned to the local hospital for treatment or received treatment without bortezomib.

All the included patients received imaging examinations such as B-ultrasound, computed tomography (CT), local magnetic resonance imaging (MRI) or positron emission tomography-computed tomography (PET-CT) before initial treatment and at the time of relapse. Extramedullary-bone related (EMB) was defined as malignant plasma cells breaking through the cortical bone, but only forming a soft tissue mass around the bone; EME was defined as a soft tissue mass formed by malignant plasma cells invading soft tissues and organs far from bone [6,13]. If the patient has both EMB and EME, the patient will be included in the EME group.

### 2.2. Treatment

All NDMM patients received a bortezomib-based regimen as first-line induction regimen. The regimens included the PD regimen (bortezomib, dexamethasone), PCD regimen (bortezomib, cyclophosphamide, dexamethasone), PAD regimen (bortezomib, doxorubicin, dexamethasone), PTD regimen (bortezomib, thalidomide, dexamethasone), and PRD regimen (bortezomib, lenalidomide, dexamethasone). The specific administration method is detailed elsewhere [14]. Autologous hematopoietic stem cell transplantation (ASCT) was used as consolidation therapy after at least partial remission (PR) was achieved after 3–4 courses of induction therapy according to the patient’s age, physical status and willingness. Patients who received ASCT after induction therapy received 2–4 courses of the original treatment as consolidation therapy. After induction therapy with or without ASCT, patients receive maintenance therapy with a bortezomib, lenalidomide, or thalidomide-based regimen.

### 2.3. Efficacy assessment

The treatment efficacy of patients was assessed by IMWG criteria, including complete remission (CR), very good partial remission (VGPR), PR, stable disease (SD) and progressive disease (PD) [15,16]. Overall survival (OS) was defined as the time from the beginning of the first course of treatment to the death of the patient or the end of follow-up. For patients with relapse, OS was defined as the period from disease relapse to death or the final follow-up.

### 2.4. Data Acquisition

All patients were hospitalized during initial diagnosis and treatment, and most patients received PET-CT examination, local MRI or CT and B-ultrasound (liver, gallbladder and spleen, pancreas, retroperitoneum, renal ureter and bladder, including prostate in males, and uterus and accessories in females)) before the initial treatment and at the time of relapse. Spleen enlargement was categorized by a spleen length diameter >12.0cm, thickness >4.0cm, a splenic portal vein diameter >0.8cm under B-ultrasound examination, any line of the spleen >12cm, or the presence of 5 rib units in the cross-sectional image (the width of adjacent ribs and intercostal space, respectively, represent one rib unit) under CT or PET/CT examination. At the time of diagnosis, most patients received fluorescence in situ hybridization (FISH) to detect specific chromosomal abnormalities in bone marrow MM cells, including 1q21 gain/amplification, 17p13 deletion, 13q/13q14 deletion, and IGH translocation according to the wishes of patients and their families. A small proportion of patients with IGH translocation were tested for specific translocations, including t (4; 14), t (11; 14), and t (14; 16). The above imaging results and the patient’s Durie-Salmon (D-S) stage, international staging system (ISS), bone marrow examination results and hematuria test results can be obtained from the patient’s inpatient medical record system. From the beginning of treatment to the end of the follow-up of this study, all patients could be informed of their condition and survival through inpatient data, outpatient or telephone follow-up.

### 2.5. Statistical Analysis

All patients were followed up until 30 June 2021. The threshold of acquiring data was based on the literature, the normal threshold value of our center or the receiver operating characteristic (ROC) curve. Nonnormally distributed data were expressed as medians and compared using the Mann–Whitney U test; count data were expressed as percentages and compared using the chi-square test or Fisher’s exact test. The Kaplan–Meier method was used to generate survival curves, and the difference between the curves was compared using the log-rank test. A Cox proportional hazards model and logistic regression analysis were used to perform univariate and multivariate analyses, which were displayed as hazard ratios (HRs), odds ratios (ORs) and 95% confidence intervals (CIs). All the test results were bilateral. A *p* value < 0.05 indicated statistical significance, and factors with a *p* value < 0.1 were entered into multivariate analysis. The statistical analyses were performed using SPSS for Windows 26.0 and GraphPad Prism 8.0.

## 3. Results

### 3.1. Clinical Characteristics of Patients with Extramedullary Relapse

**Figure 1:**
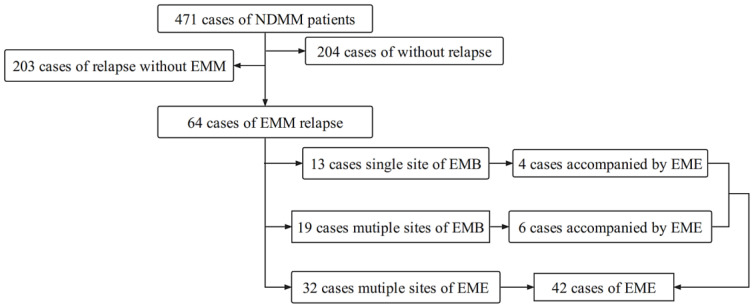
Schematic diagram of EMM patient detection.

A total of 471 NDMM patients were included in this study. During follow-up, 204 patients did not experience disease relapse or progression, and 267 patients experienced disease relapse or progression, including 64 (24.0%) patients with extramedullary relapse, among whom 22 (8.2%) patients experienced EMB relapse and 42 (15.7%) patients experienced EME relapse. Among 64 patients with extramedullary relapse, 32 patients had EMM at the time of diagnosis, and developed further extramedullary relapse during follow-up; 32 patients did not have EMM at the time of diagnosis, but developed extramedullary relapse during follow-up (Figure 1).

Compared with patients without any relapse during follow-up, patients with extramedullary relapse were more common in IgD subtype (12.7% vs. 1.5%, *p* < 0.001), more inclined to R-ISS stage 3 (45.5% vs. 29.1%, *p* = 0.025), more likely to have decreased platelet (Plt) levels (46.0% vs. 27.9%, *p* = 0.007), higher serum lactate dehydrogenase (LDH) levels (39.7% vs. 14.2%, *p* < 0.001), spleen enlargement (40.6% vs. 16.2%, *p* < 0.001), osteolytic lesions (50.0% vs. 13.2%, *p* < 0.001)and extramedullary involvement (50.0% vs. 20.6%, *p* < 0.001) (Table 1). Compared with patients with relapse but without extramedullary involvement during follow-up, patients with extramedullary relapse were more likely to have higher serum LDH levels (39.7% vs. 21.1%, *p* = 0.003), hypercalcemia (50% vs. 33.2%, *p* = 0.012), spleen enlargement (40.6% vs. 23.2%, *p* = 0.012), osteolytic lesions (50.0% vs. 15.2%, *p* < 0.001) and extramedullary involvement (50.0% vs. 17.7%, *p* < 0.001) (Table 1). 

We further analyzed the incidence of extramedullary relapse in patients with/without EMM at diagnosis, and the results showed that the incidence of extramedullary relapse in patients with EMM at diagnosis was 29.1% (32/110), which was significantly higher than that in patients without EMM at diagnosis (8.9% (32/361), *p* < 0.001). Further analysis showed that the incidence of EMB relapse in patients with EMB at diagnosis was 21.3% (20/94), which was significantly higher than that in patients without EMB at diagnosis (the incidence of EMB relapse was 11.7% (44/377), *p* = 0.015); and the incidence of EME relapse in patients with EME at diagnosis was 75% (12/16), which was significantly higher than that in patients without EME at diagnosis (the incidence of EME relapse was only 11.4% (52/455), *p* < 0.001). However, there was no significant correlation between the previous treatment regimen in the early stage, such as bortezomib-based treatment regimen and ASCT, and the occurrence of extramedullary relapse in NDMM patients (*p* > 0.005) (Table 1).

### 3.2. Effect of Extramedullary Relapse on the Prognosis of MM Patients

**Figure 2:**
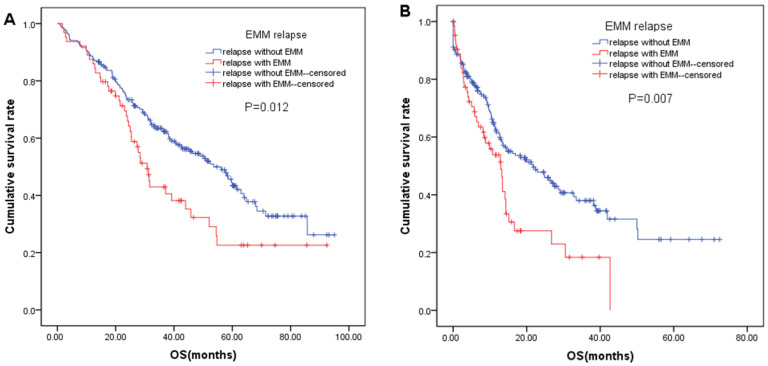
Overall survival (OS) of patients with EMM relapse and relapse without EMM (**A**); OS of relapsed patients (**B**).

According to the time of EMM occurrence, patients were divided into without EMM, EMM only at diagnosis, EMM only at relapse, and EMM at both diagnosis and relapse, and the median OS of the four groups were 85.7 months, not reached (NR), 45.8 months (95%CI: 29.9–61.7), and 25.0 months (95%CI: 19.7–30.2), respectively (*p* < 0.01) (Supplementary Figure S1). During follow-up, 267 patients had disease relapse or progression, of which 203 patients had disease relapse without extramedullary involvement, and 64 patients had extramedullary relapse. We compared the median OS of 267 relapsed patients with or without extramedullary involvement, and survival analysis indicated that compared with patients with disease relapse without extramedullary involvement, extramedullary relapse patients had significantly worse median OS. The median OS was 30.8 months (95% CI: 26.6–35.0) and 53.6 months (95% CI: 63.5–63.8), respectively, and the 3-year OS rates were 42.9% and 62.9%, respectively (Figure 2A). In addition, we further compared and analyzed the difference in the median OS of patients after disease relapse and progression, and survival analysis showed that the median OS of patients with extramedullary relapse was significantly worse than that of patients with relapse without extramedullary involvement (*p* = 0.007). The median OS was 12.9 months (95% CI: 9.1–16.8) and 27.7 months (95% CI: 13.2–30.3), respectively, and the 3-year OS rates were 18.4% and 36.3%, respectively (Figure 2B). 

### 3.3. Risk Factors for Extramedullary Relapse in NDMM Patients

**Figure 3:**
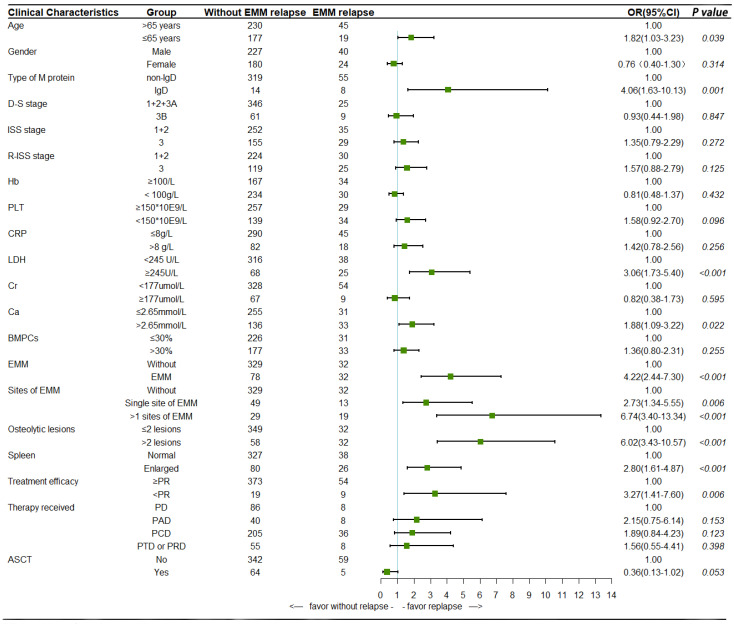
Univariate analysis of risk factors for extramedullary relapse in newly diagnosed MM patients.

We further analyzed the risk factors for extramedullary relapse in NDMM patients, and performed univariate and multivariate logistic regression analyses on 471 NDMM patients. Univariate analysis suggested that younger age (<65 years, OR = 1.82, 95% CI:1.03–3.23, *p* = 0.039), type of M protein (OR = 4.06, 95% CI:1.63–10.13, *p* = 0.001), elevated LDH (OR = 3.06, 95% CI: 1.73–5.40, *p* < 0.001), hypercalcemia (OR = 1.88, 95% CI: 1.09–3.22, *p* = 0.022), ˃2 osteolytic lesions(OR = 6.02, 95% CI: 3.43–10.57, *p* < 0.001), spleen enlargement (OR = 2.80, 95% CI: 1.61–4.87, *p* < 0.001), extramedullary involvement (OR = 4.22, 95% CI: 2.44–7.30, *p* < 0.001) and poor treatment efficacy (<PR, OR = 3.27, 95% CI: 1.41–7.60, *p* = 0.001) at the time of MM diagnosis were risk factors for extramedullary relapse in NDMM patients (Figure 3).The above factors were included in multivariate analysis, and the results confirmed that elevated LDH (OR = 2.09, 95% CI: 1.11–3.96, *p* = 0.023), ˃2 osteolytic lesions (OR = 3.70, 95% CI: 1.99–6.89, *p* < 0.001), spleen enlargement (OR = 2.27, 95% CI: 1.21–4.26, *p* = 0.011) and extramedullary involvement (OR = 3.48, 95% CI: 1.89–6.40, *p* < 0.001) at the time of MM diagnosis were independent risk factors for extramedullary relapse in NDMM patients.

**Figure 4:**
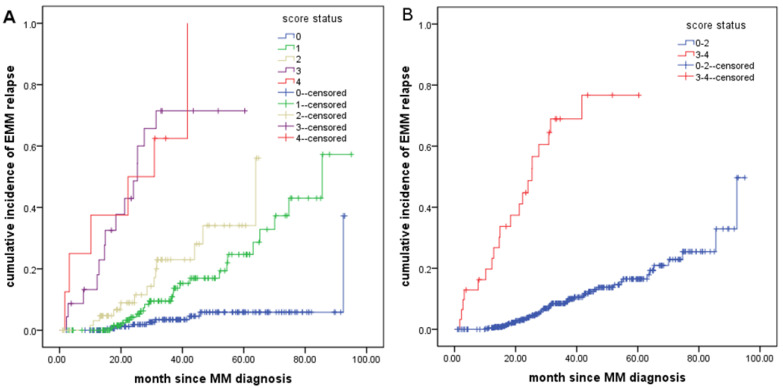
The incidence of extramedullary relapse in newly diagnosed MM patients under different score statuses (**A**,**B**).

Serum LDH ≥245U/L, ˃2 osteolytic lesions, spleen enlargement and extramedullary involvement at the time of diagnosis were assigned a value of 1 to form the extramedullary relapse prediction score. The results indicated that 194 patients had a score of 0, 155 patients had a score of 1, 68 patients had a score of 2, 23 patients had a score of 3, and 8 patients had a score of 4. There were 8 (4.1%), 23 (14.8%), 13 (19.1%), 14 (60.9%) and 6 (75.0%) patients with extramedullary relapse in the 5 groups, respectively, and the 3-year extramedullary relapse rates of the 5 groups of patients were 4.6%, 10.9%, 28.1%, 71.5%, 100%, respectively (Figure 4A, Supplementary Table S1). The patients were further divided into 0–2 points and 3–4 points groups, and patients in the 0–2 points group was defined as low-risk extramedullary relapse group, and the 3–4 points group was defined as high-risk extramedullary relapse group. The 3-year extramedullary relapse rates of the two groups of patients were 9.0% and 76.7%, respectively, and there was a significant difference in the extramedullary relapse rates between the two groups (*p* < 0.001) (Figure 4B), which indicates that the extramedullary relapse prediction score model can effectively predict the high-risk extramedullary relapse population in NDMM patients.

To examine the role of the extramedullary relapse prediction score model in predicting extramedullary relapse in patients with NDMM, we further analyzed the predictive ability of existing ISS stage and R-ISS stage for extramedullary relapse in NDMM patients. The results showed that extramedullary relapse occurred in 17 patients (12.3%) with ISS stage I, 18 patients (12.1%) with ISS stage II and 29 patients (15.8%) with ISS stage III (*p* = 0.544), and the 3-year extramedullary relapse rates in the three groups were 8.8%, 11.3% and 23.5%, respectively (Supplementary Table S1, Supplementary Figure S2). Extramedullary relapse occurred in 4 patients (8.2%) in R-ISS stage I, 26 patients (12.7%) in R-ISS stage II and 25 patients (17.5%) in R-ISS stage III (*p* = 0.214), and the 3-year extramedullary relapse rates in the three groups were 3.2%, 12.1% and 21.6%, respectively (Supplementary Table S1, Supplementary Figure S2).

## 4. Discussion

MM is still an incurable disease, and the majority of patients will eventually relapse, which manifests as the reappearance of M protein in blood or urine, increased bone marrow plasma cell percentage, hypercalcemia, new osteolytic lesions or soft tissue plasmacytoma. Extramedullary relapse is not uncommon in MM patients and is estimated to account for approximately 10–20% of MM relapses according to the literature [17]. In recent years, due to the progress of treatment and the increased sensitivity of imaging methods, the proportion of patients with extramedullary relapse has increased significantly, and many studies have confirmed that extramedullary relapse is significantly associated with high-risk clinical characteristics and poor prognosis. Varettoni et al. demonstrated that patients with extramedullary relapse had significantly lower serum M protein (1.6 g/dL vs. 3.3 g/dL, *p* = 0.02) and hemoglobin levels (11.5 g/dL vs. 12.7 g/dL, *p* = 0.02), and higher LDH levels (550 U/L vs. 314 U/L, *p* = 0.009) [12]. Pour et al. confirmed that extramedullary relapse was associated with poor prognosis, and patients with extramedullary relapse had a significantly shorter median OS than patients without extramedullary relapse (38 months vs. 109 months, *p* < 0.001). Moreover, patients with EME relapse had significantly worse median OS than patients with EMB relapse (30 months vs. 45 months, *p* = 0.022) [10]. Stork et al. also demonstrated that NDMM patients with extramedullary relapse had significantly inferior median PFS (13.8 months vs. 18.8 months, *p* = 0.006) and OS (26.7 months vs. 58.7 months, *p* < 0.001) compared with NDMM patients without extramedullary relapse during follow-up, and multivariate analysis confirmed that extramedullary relapse was an independent prognostic risk factor for PFS and OS in refractory/relapsed MM (RRMM) patients [9].

At present, EMM patients mostly follow the treatment of MM patients, and adopt a variety of drug combination schemes with different mechanisms [6]. However, the prognosis of EMM patients is usually very poor, especially for patients with extramedullary relapse, which remains a major challenge for the treatment of MM patients even in the era of new drugs [8,12,18,19]. The standard RRMM treatment regimen based on proteasome inhibitors and immunomodulators does not significantly improve the prognosis of extramedullary relapse patients [9,11,12,20] because some studies believe that thalidomide and bortezomib can induce the dedifferentiation of bone marrow plasma cells and alter the expression of adhesion molecules, allowing myeloma clones to escape from the marrow microenvironment and thus facilitating extramedullary spread [21]. Although the second-generation proteasome inhibitor carfizomib has certain efficacy in patients with extramedullary relapse, it still cannot overcome its negative effects [22]. Although ASCT is considered to improve the prognosis of EMM patients, studies have found that the extramedullary relapse rate after ASCT and allo-SCT was higher than before, up to 32–35% [23,24,25,26]. Analysis of the reasons may be because tumor plasma cells surviving after ASCT can lose cell-to-cell interactions in the bone marrow microenvironment, making it easier to diffuse and infiltrate into other sites, resulting in an increased incidence of extramedullary relapse [26]. Monoclonal antibodies, such as CD38 and signaling lymphocytic activation molecule family member 7 (SLAMF7), targeting cell surface markers, and have significant efficacy in MM patients [27,28,29]. Due to the decreased expression of CD38 in plasma cells of EMM patients, studies have confirmed that the efficacy of daratumumab in EMM patients is limited, and the overall response rate (ORR) of monotherapy is only 17% [30,31]. Elotuzumab is the first approved SLAMF7 monoclonal antibody for the treatment of RRMM patients [28], but unfortunately, detailed treatment response and outcome data for EMM patients have not been fully reported to date. Recent studies by Danhof et al. have demonstrated that elotuzumab-based combination therapies had limited efficacy in EMM patients, with an ORR of only 40%, and the median PFS and OS was 3.8 and 12.9 months, respectively [32]. Although chimeric antigen receptor T cell (CAR-T) immunotherapy has shown good results in EMM patients, further clinical studies are still needed to evaluate its short-term and long-term effects [32,33].

Due to the lack of prospective clinical studies, the risk factors for extramedullary relapse have not been fully elucidated. An earlier study found that patients with multiple sites of osteolytic lesions at diagnosis had a higher incidence of extramedullary relapse (62.5% vs. 37.5%), and extramedullary relapse patients were relatively younger (60 vs. 63 years, *p* = 0.073), and more commonly diagnosed with the IgA (25.8% vs. 20.8%) and non-secretory types of MM (2.2% vs. 0) [34]. Stork et al. retrospectively analyzed the risk factors for extramedullary relapse in NDMM patients and found that younger age (<65 years; OR = 4.38, *p* < 0.0001), higher LDH levels (>5 μkat/L; OR = 2.07, *p* < 0.0001), >2 osteolytic lesions (OR = 2.21, *p* < 0.001), and IgA (OR = 1.53, *p* = 0.009) or nonsecretory type of MM (OR = 2.83, *p* = 0.007) at the time of MM diagnosis were the main risk factors for extramedullary relapse in NDMM patients [9]. To further understand the clinical characteristics of the high-risk of extramedullary relapse in NDMM patients, we analyzed the high-risk factors for extramedullary relapse in 471 NDMM patients. Multivariate analysis confirmed that elevated LDH (OR = 2.09, *p* = 0.023), >2 osteolytic lesions (OR = 3.70, *p* < 0.001), extramedullary involvement (OR = 3.48, *p* < 0.001) and splenomegaly (OR = 2.27, *p* = 0.011) at the time of MM diagnosis were independent risk factors for extramedullary relapse in NDMM patients. To better predict high-risk extramedullary relapse in NDMM patients, we assigned the above four risk factors 1 point to establish an extramedullary relapse prediction score model, which showed that the 3-year extramedullary relapse rates in low-risk and high-risk patients were 9.0% and 76.7%, respectively. Although there is a difference in the probability of extramedullary relapse within 3 years between ISS and R-ISS stages, which has a certain role in indicating extramedullary relapse, the distinction is not obvious, especially in high-risk (stage 3) patients in whom the probability of extramedullary relapse is less than 25%. However, the 3-year extramedullary relapse rates of high-risk patients with the extramedullary relapse prediction score established by us are as high as 76%, which has a better ability to predict extramedullary relapse than the existing ISS stage and R-ISS stage. Accurate survival prediction is important because prognostic factors influence treatment choice [35]. Early identification of patients with high-risk extramedullary relapse and taking active treatment measures to prevent the emergence of extramedullary relapse in clinical practice is of great significance for improving the prognosis of NDMM patients. To improve the prognosis of patients with high-risk extramedullary relapse, we recommend that NDMM patients with high-risk extramedullary relapse adopt stronger and more optimized treatment strategies at the initial diagnosis to prevent early extramedullary relapse, such as induction therapy with different mechanism drugs, followed by routine double ASCT and triple consolidation therapy and maintenance therapy [36,37,38].

Our study found for the first time that spleen morphological enlargement is a poor prognostic factor for extramedullary relapse. However, since the definition of spleen enlargement in our article is mainly based on imaging findings, in the absence of pathological results, it is difficult to determine whether spleen enlargement is due to extramedullary involvement, amyloidosis, splenic blood stasis, or combined with other hereditary metabolic cells (Gaucher Disease) and many other unexplained causes [39], which might have some certain impact on the reliability of the data, and further research is still needed. In addition, this study was a retrospective study, patients only received bortezomib-based treatment, and only a small number of patients received the immunomodulator thalidomide/lenalidomide, which is not the currently recommended combination of multiple new drugs with different mechanisms, and the proportion of patients receiving ASCT is low. Therefore, it is still necessary to further confirm the role of the scoring system in NDMM patients in a large prospective multicenter study, and to design corresponding treatment measures for the selected high-risk extramedullary relapse patients to prevent the occurrence of extramedullary relapse to further clarify whether this scoring system can improve the prognosis of high-risk extramedullary relapse patients.

## 5. Conclusions

Our study confirmed that extramedullary relapse was associated with high-risk clinical characteristics and poor prognosis among patients treated with bortezomib-based regimens. Therefore, in clinical practice, it is necessary to identify patients with high-risk extramedullary relapse early, and take active treatment measures to improve the prognosis of NDMM patients. Elevated LDH, >2 osteolytic lesions, extramedullary involvement and enlarged spleen at the time of MM diagnosis were independent risk factors for extramedullary relapse in NDMM patients, and the extramedullary relapse prediction model composed of the above four factors had a better ability to predict extramedullary relapse than the existing ISS and R-ISS stages.

## Figures and Tables

**Table 1:** Clinical characteristics of EMM patients (N = 471).

	Without Relapse N = 204(%)	Relapse Without EMM N = 203(%)	Relapse With EMM N = 64(%)	*p* Value ^1^	*p* Value ^2^
Age				0.075	0.037
≤65 years	118 (57.8)	112 (55.2)	45 (70.3)		
>65 years	86 (42.2)	91 (44.8)	19 (29.7)		
Gender				0.386	0.302
Male	115 (56.4)	112 (55.2)	40 (62.5)		
Female	89 (43.6)	91 (44.8)	24 (37.5)		
Type of M protein, n (%)				<0.001	0.095
Non-IgD	200 (98.5)	191 (94.6)	55 (87.3)		
IgD	3 (1.5)	11 (5.4)	8 (12.7)		
D-S stage, n (%)				0.207	0.915
1 + 2	56 (27.5)	39 (19.2)	11 (17.2)		
3A	117 (57.4)	134 (66.0)	14 (68.8)		
3B	31 (15.2)	30 (14.8)	9 (14.1)		
ISS, n (%)				0.510	0.639
1	64 (31.4)	57 (28.1)	17 (26.6)		
2	64 (31.4)	67 (33.0)	18 (28.1)		
3	76 (37.3)	79 (38.9)	29 (45.3)		
R-ISS, n (%)				0.025	0.516
1 + 2	124 (70.9)	100 (59.5)	30 (54.5)		
3	51 (29.1)	68 (40.5)	25 (45.5)		
Unknow	29	30	14		
Hb (g/L), median(range)	94 (49–160)	89 (42–161)	99 (49–159)	0.841	0.100
≥100	96 (51.8)	71 (35.1)	34 (53.1)	0.849	0.092
<100	103 (51.8)	131 (64.9)	30 (46.9)		
Plt (×10^9^/L), median(range)	198 (31–597)	158 (36–397)	155 (23–354)	0.002	0.870
≥150 × 10	142 (72.1)	115 (57.8)	34 (54.0)	0.007	0.594
<150 × 10	55 (27.9)	84 (42.2)	29 (46.0)		
CRP (g/L), median(range)	1.8 (0–34.2)	2.2 (0–8.1)	2.1 (0–20.4)	0.282	0.865
≤8	152 (81.2)	139 (47.7)	45 (71.4)	0.102	0.606
>8	35 (18.8)	47 (25.3)	18 (28.6)		
LDH (U/L), median(range)	171.5 (85–605)	188 (79–848)	218 (83–5785)	<0.001	0.012
<245	163 (85.5)	156 (78.9)	38 (60.3)	<0.001	0.003
≥245	27 (14.2)	41 (21.1)	25 (39.7)		
Ca^2+^ (mmol/L)					
≤2.65	124 (63.6)	131 (66.8)	31 (50.0)	0.057	0.017
>2.65	71 (36.4)	65 (33.2)	31 (50.0)		
BMPCs (%), median(range)	22.0 (0–96)	31.0 (0–97)	30.8 (1–99)	0.003	0.954
≤30	128 (63.4)	98 (48.8)	31 (48.4)	0.034	0.965
>30	74 (36.6)	103 (51.2)	33 (51.6)		
EMM at diagnosed				<0.001	<0.001
Non-EMM	162 (79.4)	167 (82.3)	32 (50.0)		
EMM	42 (20.6)	36 (17.7)	32 (50.0)		
EMB	40 (19.6)	34 (16.7)	20 (31.3)		
EME	2 (1.0)	2 (1.0)	12 (18.8)		
Osteolytic lesions				<0.001	<0.001
≤2 lesions	177 (86.8)	172 (84.7)	32 (50.0)		
>2 lesions	27 (13.2)	31 (15.2)	32 (50.0)		
Spleen				<0.001	0.006
Normal	171 (83.3)	156 (76.8)	38 (59.4)		
Enlarged	33 (16.2)	47 (23.2)	26 (40.6)		
Treatment efficacy				<0.001	0.334
≥PR	200 (100)	173 (90.1)	54 (85.7)		
<PR	0	19 (9.9)	9 (14.3)		
Therapy received				0.286	0.234
PD	38 (19.7)	48 (24.9)	8 (13.3)		
PAD	19 (8.8)	23 (11.9)	8 (13.3)		
PCD	99 (51.3)	106 (54.9)	36 (60.0)		
PTD or PRD	39 (20.2)	16 (8.3)	8 (13.3)		
ASCT				0.008	0.280
No	161 (79.3)	181(89.2)	60 (93.8)		
Yes	42 (20.7)	22 (10.8)	4 (6.3)		

^1^ EMM relapse compared with without relapse; ^2^ EMM relapse compared with relapse without EMM. Abbreviation: EMM, extramedullary multiple myeloma; D-S, Durie-Salmon staging; ISS, International Staging System; R-ISS, Revised International Staging System; Hgb, hemoglobin; Plt, platelet; CRP, C-reaction protein; LDH, lactate dehydrogenase; Cr, creatinine; BMPCs, bone marrow plasma cell percentages; EMB, extramedullary-bone related; EME, extramedullary extraosseous; PR, partial remission; PD, bortezomib, dexamethasone; PCD, bortezomib, dexamethasone, cyclophosphamide; PAD, bortezomib, dexamethasone, adriamycin; PTD, bortezomib, dexamethasone, thalidomide; PRD, bortezomib, dexamethasone, lenalidomide; ASCT, autologous hematopoietic stem cell transplantation.

## Data Availability

The datasets used and/or analyzed during the current study are available from the corresponding author on reasonable request.

## Supplementary Materials

The following supporting information can be downloaded at: https://www.mdpi.com/article/10.3390/cancers14246106/s1, Table S1: comparison of extramedullary relapse prediction ability of different models. Figure S1: overall survival (OS) of patients without EMM at diagnosis and relapse, EMM only at diagnosis, EMM only at relapse and EMM at both diagnosis and relapse. Figure S2: the incidence of extramedullary relapse in newly diagnosed MM patients under different ISS (A) and R-ISS stage (B).
