# Supplementary material for: Analysis of High-Risk Extramedullary Relapse Factors in Newly Diagnosed MM Patients
Source: Cancers (Basel). 2022 Dec 12;14(24):6106. doi: 10.3390/cancers14246106 (PMC9776506; doi:10.3390/cancers14246106)

Supplementary Table S1 Comparison of extramedullary relapse prediction ability of different models

Group	ISS (%)			R-ISS (%)			Extramedullary relapse prediction score (%)				
	1 (N=138)	2 (N=149)	3 (N=184)	1 (N=49)	2 (N=205)	3 (N=144)	0 (N=194)	1 (N=155)	2 (N=68)	3 (N=23)	4 (N=8)
EMM relapse	17(12.3)	18(12.1)	29(15.8)	4(8.2)	26(12.7)	25(17.5)	8(4.1)	23(14.8)	13(19.1)	14(60.9)	6(75.0)
Without EMM relapse	121(87.7)	131(87.9)	155(84.2)	45(91.8)	179(87.8)	119(82.6)	186(95.6)	132(85.2)	55(80.9)	9(39.1)	2(35.0)
P value	0.544			0.214			<0.001				
3-year EMM relapse rates	8.8	11.3	23.5	3.2	12.1	21.6	4.6	10.9	28.1	71.5	100
P value	0.039			0.030			<0.001				

Supplementary Figure S1 Overall survival (OS) of patients without EMM at diagnosis and relapse, EMM only at diagnosis, EMM only at relapse and EMM at both diagnosis and relapse.

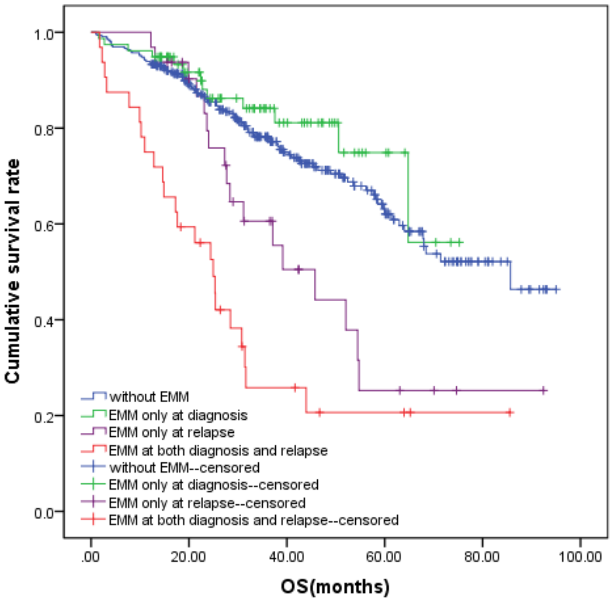

Supplementary Figure S2 The incidence of extramedullary relapse in newly diagnosed MM patients under different ISS (A) and R-ISS stage (B)

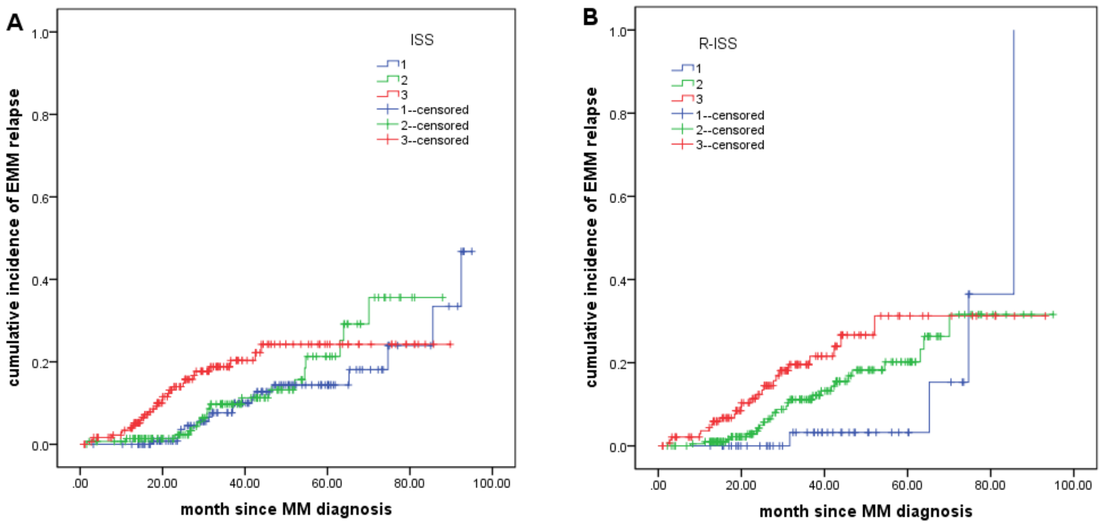

Supplement: Supplementary file 1 [file cancers-14-06106-s001.zip › cancers-2024183-supplementary.pdf]
